# Inflammatory bowel disease and the risk of intracerebral hemorrhage: A Mendelian randomization study and meta‐analysis

**DOI:** 10.1002/iid3.1048

**Published:** 2023-10-17

**Authors:** Yanju Song, Xuelun Zou, Yi Zeng, Le Zhang, Xinfa Mao

**Affiliations:** ^1^ Department of Neurology Changsha Third Hospital Changsha Hunan China; ^2^ Department of Neurology, Xiangya Hospital Central South University Changsha Hunan P.R. China; ^3^ Department of Geriatrics, Second Xiangya Hospital Central South University Changsha Hunan P.R. China; ^4^ National Clinical Research Center for Geriatric Disorders, Xiangya Hospital Central South University Changsha Hunan P.R. China; ^5^ Multi‐Modal Monitoring Technology for Severe Cerebrovascular Disease of Human Engineering Research Center, Xiangya Hospital Central South University Changsha Hunan P.R. China

**Keywords:** Crohn's disease, genome‐wide association analysis, inflammatory bowel disease, intracerebral hemorrhage, Mendelian randomization, ulcerative colitis

## Abstract

**Background:**

The link between inflammatory bowel disease (IBD) and intracerebral hemorrhage (ICH) is still unclear.

**Aims:**

We conducted a Mendelian randomization research and meta‐analysis to explore the impact of IBD and its subtypes (Crohn's disease [CD], ulcerative colitis [UC]) on the risk of ICH.

**Methods:**

Two large genome‐wide association analysis studies of International Inflammatory Bowel Disease Genetics Consortium (IIBDGC) and International Stroke Genetics Consortium as exposure (IBD, UC, and CD) and outcome (ICH) in the initial stage. IBD, CD, UC GWAS data from the FinnGen consortium were adopted for the replication phase, and ultimately, the results of the initial stage and replication phase data were combined in a meta‐analysis to evaluate the causal association between IBD and its subtypes and the risk of ICH.

**Results:**

In the initial stage, we found that in the IVW (odds ratio [OR] = 0.83, 95% confidence interval [CI]: 0.71–0.96, *p* = .01), MR‐PRESSO (OR = 0.85, 95% CI: 0.75–0.97, *p* = .02) and MR.RAPS (OR = 0.86, 95% CI: 0.76–0.98, *p* = .02) method showed that UC is associated with the risk of ICH. The causal relationship between IBD, CD, and the risk of ICH cannot be found by the IVW method. IBD and its subtypes UC, CD, and risk of ICH cannot find the presence of heterogeneity and pleiotropy. In replication stage, IBD (OR = 0.74, 95% CI: 0.59–0.94, *p* = .0135) related to ICH, while the IVW approach did not establish a causal link in UC and CD. The meta‐analysis still indicated that UC (OR = 0.83, 95% CI: 0.72–0.93, *p* < .05) would lessen the risk of ICH while the causality between IBD, CD, and ICH was unable to be established.

**Conclusion:**

UC was causally related to ICH, but IBD and CD are not associated with ICH. The precise pathophysiological mechanism needs to be thoroughly investigated in more detail.

## Graphical abstract

This study systematically investigated the causal relationship between IBD, CD, UC, and the risk of ICH for the first time. Our findings confirmed a potential causal relationship between UC and ICH. Our findings highlighted that rs3024493, rs34920465, and rs483905 may be treated as specific biomolecular markers of ICH with potential significance in predicting the occurrence of hypertension and ICH.

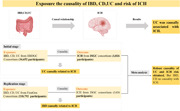

## 1 INTRODUCTION

Inflammatory bowel disease (IBD) is a prevalent autoimmune disease of the intestines, which consists of Crohn's disease (CD) and ulcerative colitis (UC). The prevalence of CD ranges from 25 to 300 cases per 100,000 individuals, and that of UC ranges from 35 to 250 cases per 100,000 people.^1^ Studies have revealed that IBD, which can afflict people of all ages and affect all bodily areas, is on the rise globally in both incidence and prevalence.^2^ Patients with IBD are susceptible to genetic factors and environmental triggers that lead to an imbalance in the intestinal flora, resulting in the disruption of the intestinal barrier.^2^, ^3^ Inflammatory cytokines, including tumor necrosis factor alpha, C‐reactive protein, interleukins, and vascular endothelial growth factor, are released into the circulation, overwhelming the immune system and leading to systemic inflammation.^3^ Inflammatory factors have the potential to penetrate the brain through the blood circulation through the blood–brain barrier, affecting the health of the cerebral vasculature. On the other hand, long‐term immune system dysregulation due to IBD may also accelerate cerebrovascular damage. Therefore, patients with IBD have a higher probability of being at risk for concurrent cerebrovascular disease.

We are gradually turning our attention to the role that the gut plays in cerebrovascular illness as a result of the thorough study of gut bacteria.^4^, ^5^ IBD, CD, and UC are all thought to be risk factors for stroke according to some meta‐analyses evaluating the relationship between these conditions and stroke.^6^, ^7^ A retrospective cohort research in Taiwan, China, involving 18,392 IBD patients and 73,568 healthy controls, revealed that IBD patients had an ischemic stroke 1.12 (95% CI: 1.02–1.23) times more frequently than individuals without IBD.^8^ However, the relationship between IBD and cerebral hemorrhage, an important subtype of stroke, is unclear.

Due to its high death and disability rates, intracerebral hemorrhage (ICH), which causes roughly 29% of strokes, poses a severe threat to human health.^9^, ^10^, ^11^ The risk factors that affect ICH are directly related to its high death rate and poor prognosis.^10^, ^12^ The dismal prognosis of ICH has not yet been improved despite steady in‐depth research on common risk factors for the disease, including hypertension, hyperlipidemia, excessive alcohol consumption, smoking, and so forth.^10^, ^11^ Therefore, early management may be able to somewhat lower the health risk of ICH if other risk factors are found.

Mendelian randomization (MR) study is a cutting‐edge epidemiological method to determine causality, which can explore the association between exposure and outcome from a genetic perspective, and discover risk factors or complications of disease, which is important for disease prevention. Therefore, to reduce the incidence of poor prognosis and prevent cerebral hemorrhage in patients with IBD, we propose to use MR studies to explore the association between IBD and its subtypes (CD and UC) and the risk of ICH. This will fill the gap in the research field of IBD and cerebrovascular disease, and make an important contribution to the accurate prediction of cerebrovascular events after reducing IBD.

## 2 METHODS

### 2.1 Research design and core assumptions

**Figure 1:**
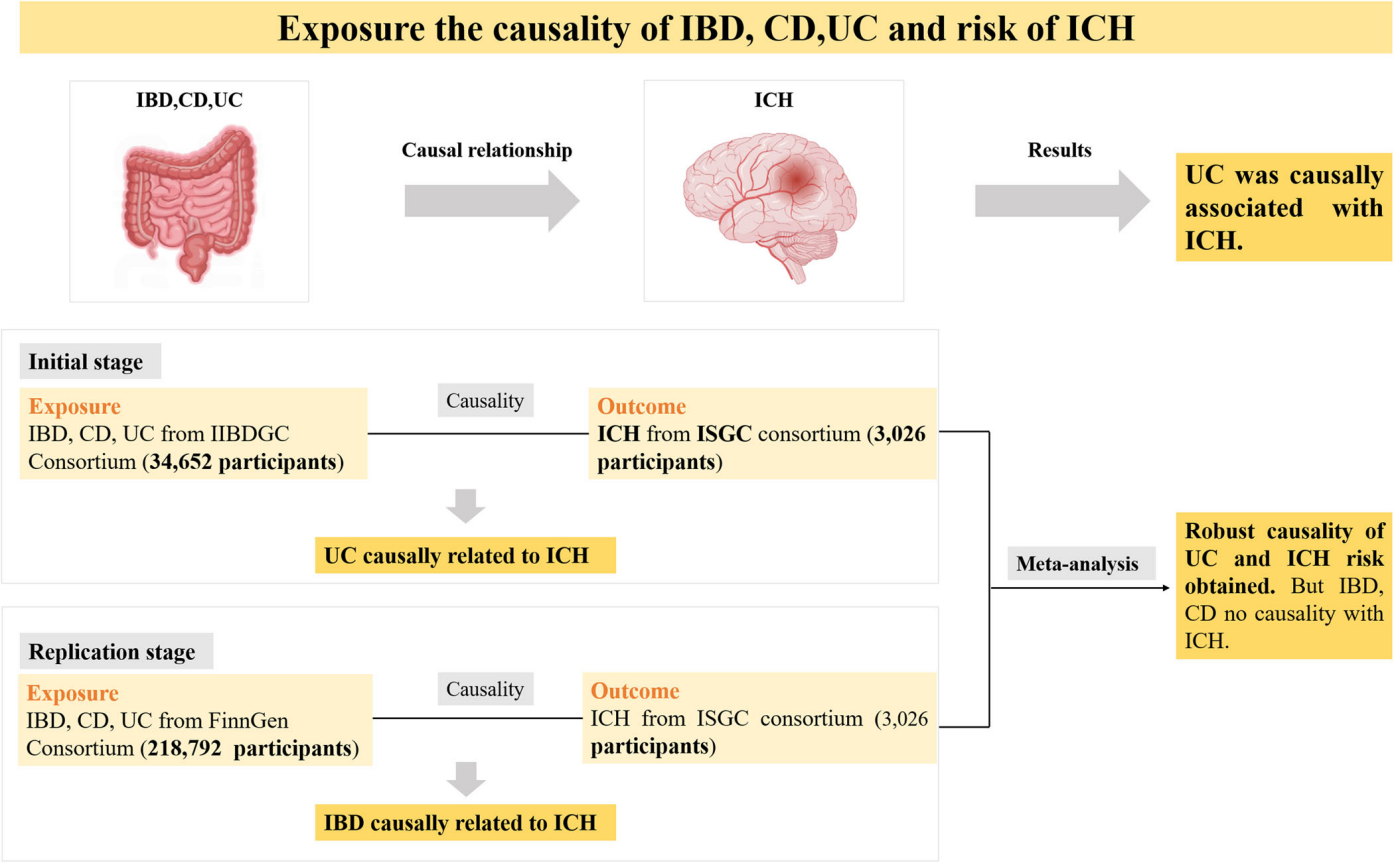
Overview of the causal connections between IBD, including CD and UC as subtypes and ICH. CD, Crohn's disease; IBD, inflammatory bowel disease; ICH, intracerebral hemorrhage; IIBDGC, International Inflammatory Bowel Disease Genetics Consortium; ISGC, International Stroke Genetics Consortium; IVW, inverse variance weighted; UC, ulcerative colitis.

**Figure 2:**
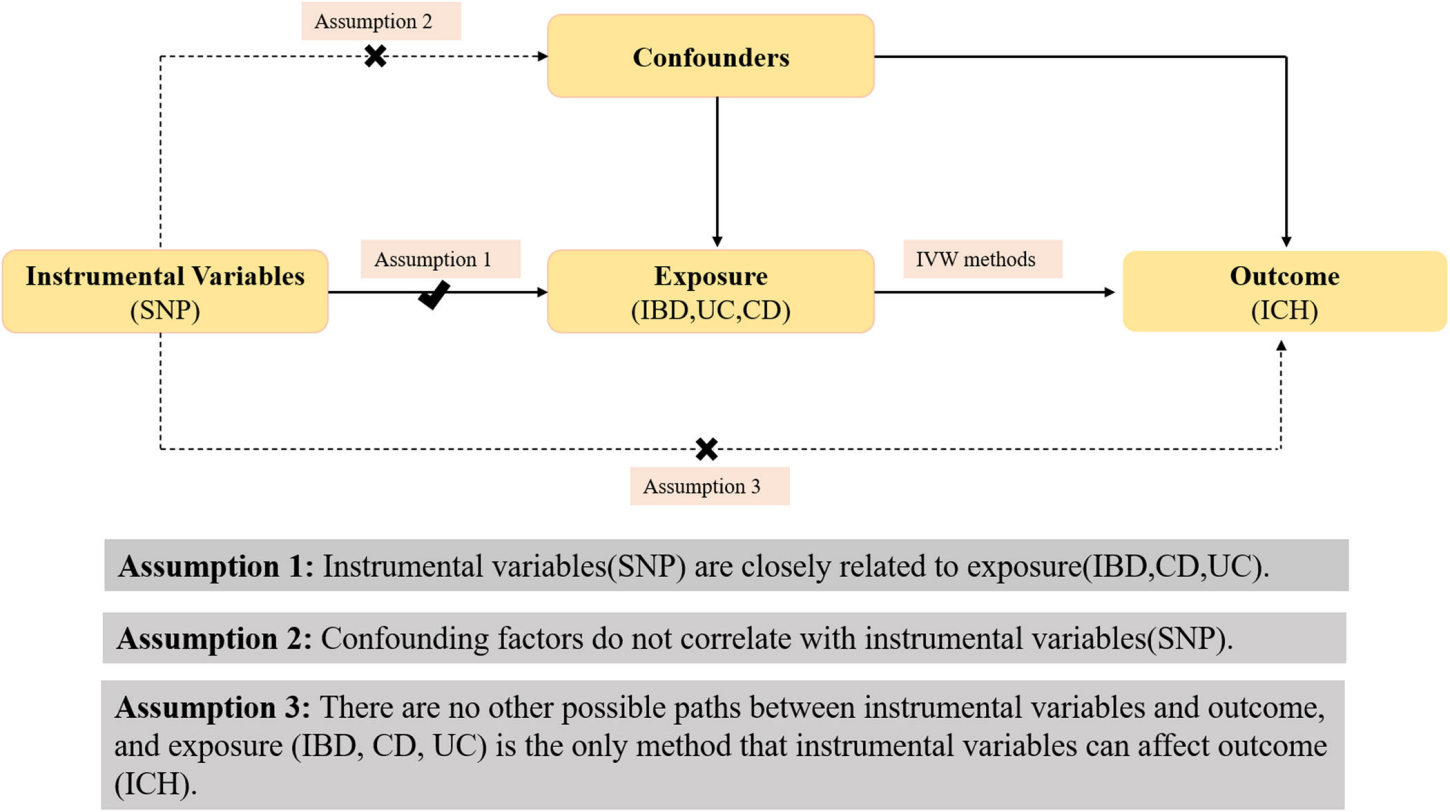
Three core assumptions of IBD, CD, UC with ICH in this two‐sample MR study. CD, Crohn's disease; IBD, inflammatory bowel disease; ICH, intracerebral hemorrhage; IVW, inverse variance weighted; UC, ulcerative colitis.

The main goal of this MR study is to establish causation through exposure, instrumental variables, and outcome. The exposure, instrumental variable, and outcome of the study were IBD and its subtypes, single‐nucleotide polymorphism (SNP) and ICH, respectively (Figure 1). Figure 2 illustrates three key assumptions that must be followed when utilizing MR in studies. First, there is a strong connection between instrumental variables (SNP) and exposure (IBD, CD, and UC). Second, there is no connection between confounders and instrumental variables (SNP). Confounding variables include smoking, binge drinking, hypertension, hypercholesterolemia, and sedentary behavior, among others. Finally, aside from exposures (IBD, CD, and UC), there are no other potential pathways through which instrumental variables (SNP) could affect the outcome (ICH).^12^, ^13^

### 2.2 Source of population

To generate the exposures and outcomes used in this investigation, a genome‐wide association study (GWAS) was conducted on a large sample of European population genomes. The GWAS data for IBD, UC, and CD were obtained from the International Inflammatory Bowel Disease Genetics Consortium (IIBDGC), according to Table 1. This GWAS study looked into the genetic risk factors for the IBD, UC, and CD clinical phenotypes. This GWAS included a total of 34,652 European populations, 12,882 IBD patients, 21,770 controls, and 12,716,084 SNP sites.^14^ It also included 27,432 individuals in UC (6968 cases and 20,464 non‐cases) and 20,883 individuals in CD (5956 cases and 14,927 non‐cases). The information on ICH was provided by a GWAS study which conducted by the International Stroke Genetics Consortium's (IGSC), with 3026 participants including 1545 cases and 1481 non‐cases.^15^ FinnGen (https://finngen.gitbook.io/documentation/data-download) provided the data for the replication phase, which included 5673 cases and 213,119 controls with a total of 16,380,466 SNPs. Data on exposures and outcome were gathered from published GWAS, and the participants gave their informed consent and underwent an ethical review. Therefore, no additional ethical review of this study is needed.

**Table 1:** Information fundamental for the inclusion of exposure and outcome data in GWAS.

Consortium	Phenotype	Number of SNP	Cases	Controls	Sample size	Population	PMID
IIBDGC	UC	12,255,197	6968	20,464	27,432	European	26192919
	CD	12,276,506	5956	14,927	20,883	European	26192919
	IBD	12,716,084	12,882	21,770	34,652	European	26192919
FinnGen	UC	16,380,453	2251	210,300	212,551	European	https://finngen.gitbook.io/documentation/data-download
	CD	16,380,466	940	217,852	218,792	European	
	IBD	16,380,466	5673	213,119	218,792	European	
ISGC	ICH	NA	1545	1481	3026	European	24656865

Abbreviations: CD, Crohn's disease; IBD, inflammatory bowel disease; ICH, intracerebral hemorrhage; SNP, single‐nucleotide polymorphism; UC, ulcerative colitis.

### 2.3 Mendelian randomization

We used the MR method to conduct this investigation, and the procedure was as follows. First, we searched the PubMed database to find the sources of the GWAS data for the included IBD, UC, CD, and ICH. Representative published GWAS data were used to generate the GWAS of exposure and outcome.^14^, ^15^ The GWAS analysis of exposure and outcome included people from the same pedigree population, as well (European population). Second, we retrieved instrumental variables for IBD, UC, and CD from the GWAS data. *p* < 5 × 10^−8^, *r* ^2^ for .01, kb = 1000 is our extraction criterion for instrumental variables. Additionally, three or more SNPs that met the inclusion requirements were considered to be able to be included as instrumental variables. When the *F*‐statistic is greater than 10, we can overlook the bias of weak instrumental variables that are assessed using this statistic.^16^ Then, using MR study methods such as inverse variance weighted (IVW), the weighted median estimate (WME), MR‐Robust Adjusted Profile Score (MR.RAPS), MR‐Pleiotropy Residual Sum and Outlier (MR‐PRESSO), and MR‐Egger regression method, the causal link between exposure and outcome was evaluated.^17^, ^18^, ^19^, ^20^, ^21^ The IVW technique, which means SNP–outcome associations divided by SNP‐exposure associations, is the dominant method for determining causality with multiplicative random effects.^17^ WME assumptions are based on 50% weights from valid SNPs.^18^ The no measurement error hypothesis is answered by the MR‐Egger regression approach, which can still find stronger causation in the presence of erroneous instrumental variables.^19^ When the intercept of MR‐Egger regression is zero, it shows that there is no genetic pleiotropy, which is another way to measure pleiotropy.^19^ A cutting‐edge technique for determining causation is MR‐PRESSO.^21^, ^22^ The MR‐PRESSO global test can also determine whether directional pleiotropy exists in addition to causality.^22^ The MR‐PRESSO outliers test may identify whether outliers are present or absent.^21^ The robust causality evaluation approach MR.RAPS prevents weak instrumental variables, systematic polymorphisms, and particular polymorphisms from interfering with causality.^20^ Finally, the outcomes of heterogeneity tests and pleiotropy validity analyses were used to assess the robustness and reliability of the causal linkages. As indicated earlier, MR‐Egger regression and the MR‐PRESSO global test were largely used to evaluate pleiotropy.^19^, ^21^ Heterogeneity was primarily found by the IVW method and MR‐Egger regression. The presence of heterogeneity or pleiotropy was taken into consideration when *p* < .05.

### 2.4 Meta‐analysis of FinnGen and IIBDGC data

To obtain reliable causal relationships, we performed a meta‐analysis of the different results obtained by MR analysis in initial stage and replication stage. The random effects model that was utilized in this meta‐analysis had a statistical significance cutoff of *p* < .05. The statistical test for heterogeneity (including subgroup analysis) was set at .05. Therefore, *p* < .05 indicates potential heterogeneity.

### 2.5 Statistical analysis

All statistical analyses for this study were carried out using the R and R studio packages “TwoSampleMR,” “Mendelian randomization,” and “MR‐PRESSO.” The statistical significance of causality for the causality evaluation in this study was established using a multiple test (Bonferroni correction) adjustment of a *p* value less than 0.0167(0.05/3). The level of statistical significance for further studies, such as the pleiotropy test and the examination of heterogeneity, was also set at .05.

## 3 RESULTS

### 3.1 Inclusion of SNP information

There were 146 SNPs included in the IBD, 84 of which were not present in the ICH (blue markers in Supporting Information: Table 1). Eight SNPs were also eliminated because they were intermediate allele frequencies (rs10917547, rs11574906, rs12141431, rs1505992, rs35730213, rs4712528, rs6927172, rs75159542). As a result, 54 SNPs were examined to IBD and the risk of ICH. The GWAS data for UC included 110 SNPs in total, of which 79 SNPs were not present in the GWAS data for ICH (marked in blue in Supporting Information: Table 2). Additionally, five SNPs—rs10917547, rs35730213, rs484356, rs9823546, and rs9891174—were eliminated from the study of the causative connection between UC and ICH risk because they were palindromic and had intermediate allele frequencies. This left only 26 SNPs. Forty‐six of the 99 SNPs that were screened from the CD data for the examination of the causal association between CD and ICH risk were not appropriate for further investigation, and were therefore excluded (blue markers in Supporting Information: Table 3). Seven SNPs, including rs11564236, rs12141431, rs12194825, rs12692254, rs7205423, rs78487399, and rs9292782, were also eliminated because they were palindromic and had intermediate allele frequencies. As a result, only 46 SNPs were used for further research. All *F*‐statistics are more than 10, as shown in Supporting Information: Tables 1, 2, 3, indicating that the impact of weak instrumental variables can be disregarded.

**Table 2:** Heterogeneity test of IBD with its subtypes and ICH.

Consortium	Exposure	Outcome	Heterogeneity Test (IVW)			Heterogeneity Test (MR‐Egger)		
			*Q*	*Q*_df	*p* Value	*Q*	*Q*_df	*p* Value
IIBCGC	UC	ICH	34.47926	24	.0765	34.51577	25	.0973
	CD		51.07060	44	.2156	51.17552	45	.2442
	IBD		69.40437	52	.0537	69.88780	53	.0599
FinnGen	UC	ICH	1.90209	4	.7538	1.79700	3	.6156
	CD		15.65299	11	.1545	15.08598	10	.1290
	IBD		3.21776	6	.7811	3.21708	5	.6666

Abbreviations: CD, Crohn's disease; IBD, Inflammatory bowel disease; ICH, intracerebral hemorrhage; SNP, single‐nucleotide polymorphism; UC, ulcerative colitis.

**Table 3:** Pleiotropy test and outliers test of IBD with its subtypes and ICH.

Consortium	Exposure	Outcome	Pleiotropy test (MR‐Egger)			Pleiotropy test (MR‐PRESSO)		
			Egger intercept	SE	*p* Value	global test	*p* Value	Outliers
IIBDGC	UC	ICH	−0.00716720	0.04495936	.8747	40.38702	.139	NA
	CD		0.00674580	0.02243727	.7651	60.29537	.254	NA
	IBD		0.01562338	0.02595979	.5499	82.15943	.050	NA
FinnGen	UC	ICH	0.02962400	0.09138500	.7671	8.78220	.635	NA
	CD		0.05110900	0.08336600	.5535	149.11262	.202	NA
	IBD		−0.00167000	0.06387100	.9802	149.11262	.019	NA

Abbreviations: CD, Crohn's disease; IBD, Inflammatory bowel disease; ICH, intracerebral hemorrhage; SNP, single‐nucleotide polymorphism; UC, ulcerative colitis.

### 3.2 Three core assumptions are satisfied

First, from Supporting Information: Table 1, we observe that instrumental variables (SNP) and exposure (IBD, CD, and UC) have a close relationship (*p* < 5 × 10^−8^). Second, we conducted individual PhenoScanner websites (http://www.phenoscanner.medschl.cam.ac.uk/) on each instrumental variable and found no confounding variables (smoking, excessive drinking, hypertension, hypercholesterolemia, and sedentary behavior) related to the instrumental variables (*p* < 1 × 10^−5^ and *r* ^2^ < 0.8). Finally, the MR‐PRESSO global test and MR‐Egger regression test did not reveal the presence of pleiotropy (Tables 2 and 3), indicating that instrumental variables only affect the findings through exposure and that there are no other possible pathways.

### 3.3 UC associates with the risk of ICH

**Figure 3:**
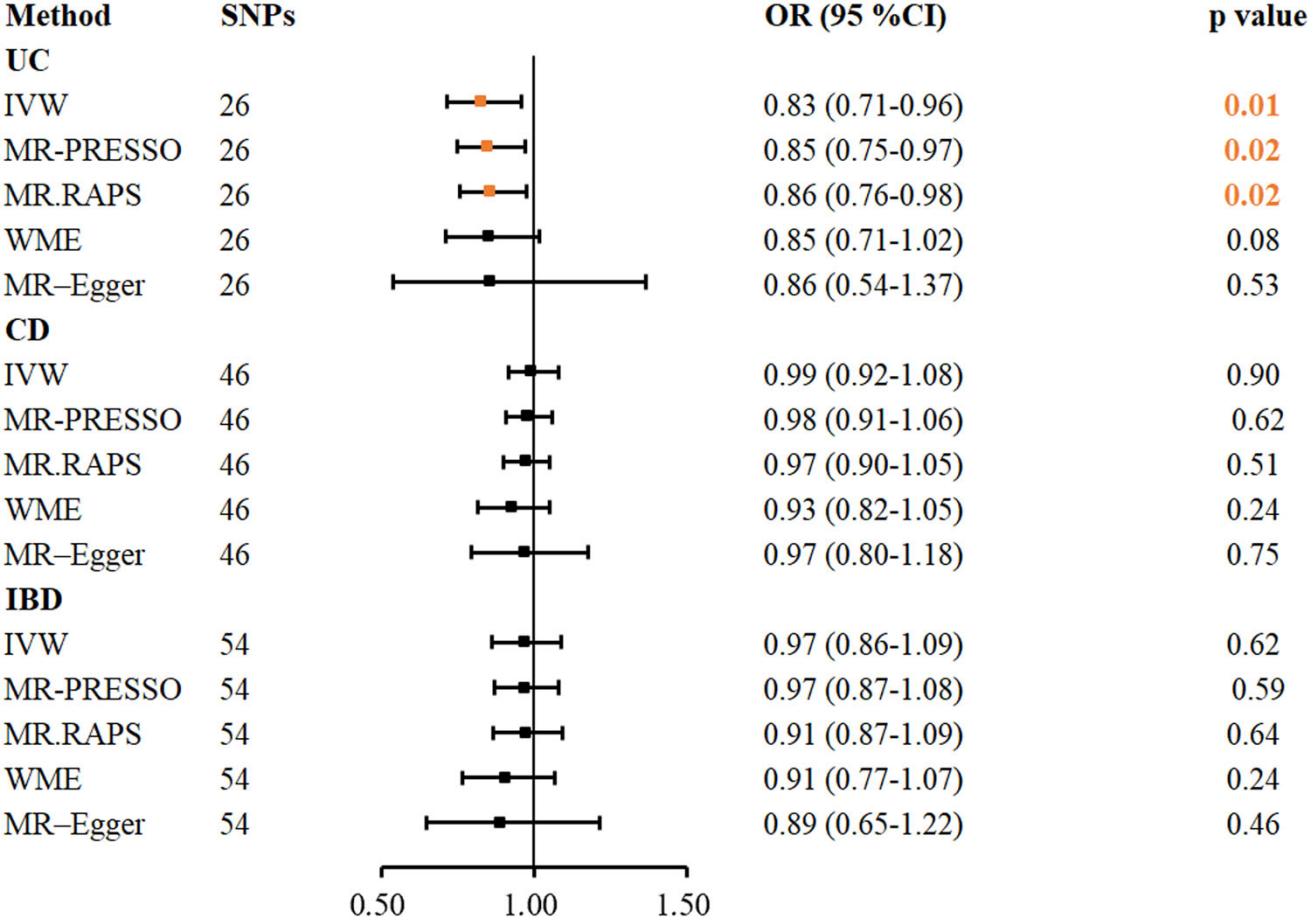
Five methods to assess the causal relationship between IBD and its UC, CD, and the risk of ICH. IBD, inflammatory bowel disease; ICH, intracerebral hemorrhage; CD, Crohn's disease; MR‐PRESSO, MR‐Pleiotropy Residual Sum and Outlier; MR.RAPS, MR‐Robust Adjusted Profile Score; UC, ulcerative colitis; WME, weighted median estimation.

The causative relationship between IBD and its subtypes and the risk of ICH was examined using MR studies, and it was found that utilizing the IVW approach, UC may reduce that risk of ICH (OR = 0.83, 95% CI: 0.71–0.96, *p* = .01) (Figure 3). The results of the MR‐PRESSO (OR = 0.85, 95% CI: 0.75–0.97, *p* = .02) and MR.RAPS (OR = 0.86, 95% CI:0.76–0.98, *p* = .02) techniques also suggest that UC may decrease the risk of ICH.

Nevertheless, the IVW method failed to find evidence of a connection between CD (OR = 0.99, 95% CI: 0.92–1.08, *p* = .90), IBD (OR = 0.97, 95% CI: 0.86–1.09, *p* = .62), and the risk of ICH. Additionally, no association between CD and ICH risk was found using the MR‐Egger regression (OR = 0.97, 95% CI: 0.80–1.18, *p* = .75), WME (OR = 0.93, 95% CI: 0.82–1.05, *p* = .24), MR‐PRESSO (OR = 0.98, 95% CI: 0.91–1.06, *p* = .62), or MR.RAPS (OR = 0.97, 95% CI: 0.90–1.05, *p* = .51). Similarly, the MR‐Egger regression (OR = 0.89, 95% CI: 0.65–1.22, *p* = .46), WME (OR = 0.91, 95% CI: 0.77–1.07, *p* = .24), MR‐PRESSO (OR = 0.97, 95% CI: 0.87–1.08, *p* = .59), and MR.RAPS (OR = 0.91, 95% CI: 0.87–1.09, *p* = .64) methods failed to show a connection between IBD and the risk of ICH.

### 3.4 Meta‐analysis of IIBDGC and FinnGen GWAS data confirm that UC related to the risk of ICH

**Figure 4:**
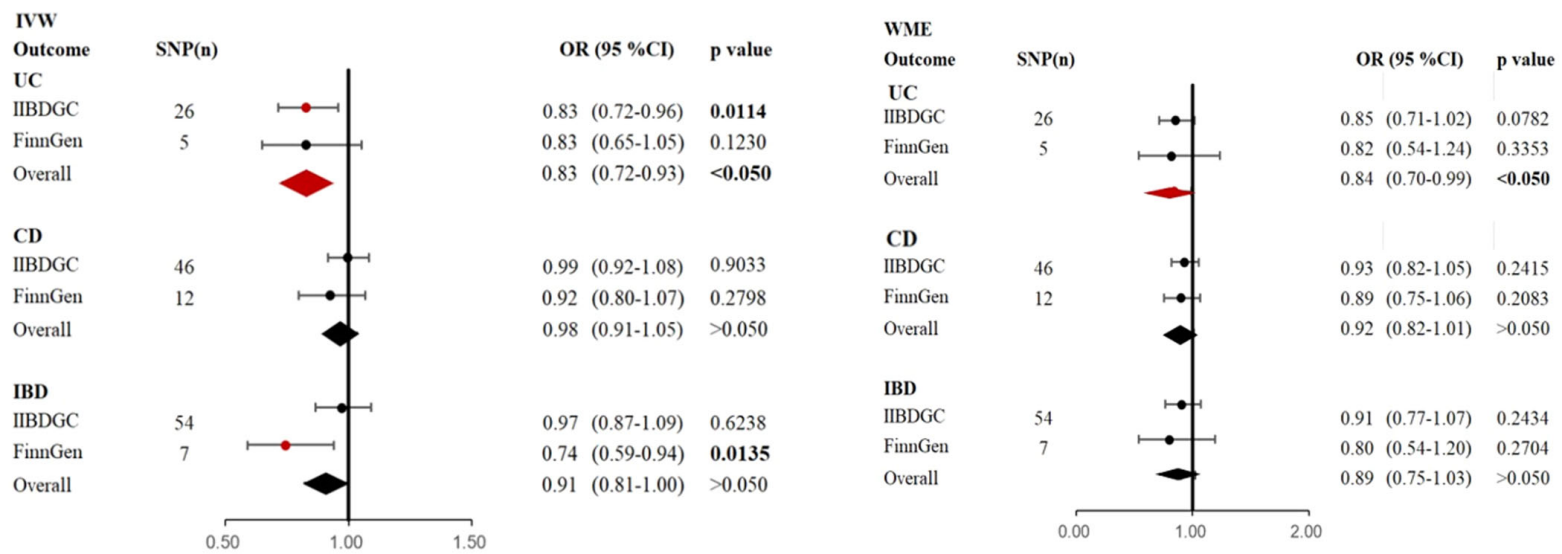
Meta‐analysis show the causality of IBD, UC, and CD with the risk of ICH. CD, Crohn's disease; IBD, inflammatory bowel disease; ICH, intracerebral hemorrhage; IIBDGC, International Inflammatory Bowel Disease Genetics Consortium; MR‐PRESSO, MR‐Pleiotropy Residual Sum and Outlier; UC, ulcerative colitis; WME, weighted median estimation.

We used FinnGen data as replication data to assess the causal relationship between IBD and its subtypes and the risk of ICH, as shown in Figure 4. We found that IBD (OR = 0.74, 95% CI: 0.59–0.94, *p* = .0135) may lower the risk of ICH; however, the IVW method did not show a causal link between UC (OR = 0.83, 95% CI: 0.65–1.05, *p* = .1230), CD (OR = 0.92, 95% CI: 0.80–1.07, *p* = .2798), and ICH. The results of our meta‐analysis of the FinnGen and IIBDGC data, however, continued to support the notion that UC (OR = 0.83, 95% CI: 0.72–0.93, *p* < .05) would reduce the risk of ICH, but we were unable to establish a causal relationship between CD (OR = 0.98, 95% CI: 0.91–1.05, *p* > .05), IBD (OR = 0.91, 95% CI: 0.81–1.00, *p* > .05), and risk of ICH.

### 3.5 Heterogeneity test and pleiotropy test show robust causality between IBD, UC, CD, and ICH risk

We searched for heterogeneity between IBD (*p*_IVW_ = .0537, *p*_MR‐Egger_ = .0599), CD (*p*_IVW_ = .2156, *p*_MR‐Egger_ = .2442), UC (*p*_IVW_ = .0765, *p*_MR‐Egger_ = .0973), and risk of ICH using IVW and MR‐Egger regression, as shown in Table 2, but we were unable to find any. Additionally, we searched for pleiotropy using the MR‐Egger regression and MR‐PRESSO global test, but once more, no overlap between the causative effects of IBD (*p*_MR‐Egger_ = .550, *p*_MR‐PRESSO_ = .050), UC (*p*_MR‐Egger_ = .875, *p*_MR‐PRESSO_ = .139), CD (*p*_MR‐Egger_ = .765, *p*_MR‐PRESSO_ = .254), and ICH was found. There are no outliers revealed using the MR‐PRESSO outlier test.

## 4 DISCUSSION

This study aims to evaluate the genetic relationship between IBD, UC, and CD and the risk of ICH using a sizable sample of GWAS data. We found that UC may reduce the risk of ICH in the initial phase, but there was no causal relationship between IBD and CD and the risk of ICH. And in the replication phase, we did not discovery a relationship between UC, CD, and ICH, but found that IBD may reduce the risk between ICH. To obtain robust results, we further performed a meta‐analysis to confirm the relationship between UC and ICH, but the relationship between IBD, CD, and ICH was not confirmed. Finally, the validity and robustness of the causative links between IBD, UC, CD, and ICH risk were confirmed by heterogeneity tests and pleiotropy analyses.

There is still uncertainty regarding the likely mechanisms linking UC to the risk of ICH. A review of a previous study in Sweden revealed a significant risk of hemorrhagic stroke during the 1–5‐year follow‐up period following discharge from a UC hospitalization of 1.45 (95% CI: 1.03–1.97).^23^ A meta‐analysis of the relationship between IBD and stroke also revealed a favorable relationship between the two (*R* = 1.29, 95% CI, 1.16–1.43), as well as a favorable relationship between UC, CD, and stroke risk.^10^ Similarly, a different meta‐analysis discovered that IBD may raise the risk of stroke. Furthermore, in a subgroup analysis, it was discovered that IBD increased the risk of stroke in both Asians and Caucasians (Asian group: OR/RR = 1.36, 95% CI: 1.05−1.23, *p* = .094; Caucasian group: OR/RR = 1.13; CI: 1.07−1.74, *p* < .001).^9^ Thus, the pathophysiological mechanisms in UC that influence the risk of ICH are connected to the mechanisms in IBD that raise the risk of stroke. High levels of pro‐inflammatory cytokines, including tumor necrosis factor, interleukin (IL)‐1, and IL‐6, are related with the primary noninfectious systemic inflammation and hypercoagulable condition that characterize parenteral symptoms of IBD.^24^, ^25^ Due to the body's protracted inflammatory condition, venous thrombotic events and pulmonary embolism are more likely to occur.^26^ Significant platelet alterations, a rise in neutrophil extracellular traps, induced endothelial dysfunction, a hypercoagulable condition, and underlying gut flora disruptions are all brought on by UC.^26^ Additionally, UC increases the risk of thrombosis by causing endothelial dysfunction, inadequate endothelium anticoagulation, and a decrease in circulating endothelial progenitor cells.^27^, ^28^ On the other hand, ICH shows pathophysiological changes that are different from ischemic stroke. Extracellular matrix breakdown, thinning of the vessel wall, and easily ruptured vessels are the main alterations of ICH, as opposed to increased thrombotic risk and endothelial dysfunction of IBD and even UC.^29^ This could be a pathway through which UC lowers the risk of ICH, but more research is required to confirm the specific mechanism.

However, there are a few limitations within this study. First of all, it should be cautiously expanded to other communities as this study was specifically focused on people of European descent. Second, despite the fact that the data for this study were taken from GWAS data, it was not possible to stratify the sample for analysis. This was because exact information about the characteristics of the population under research, such as health status, age, smoking, and alcohol use, could not be obtained. Second, the bias caused by pleiotropy is a significant flaw in MR investigations. It is challenging to rule out the idea that any SNP in our research may, in addition to having an effect on UC, also have an impact on the risk of cerebral hemorrhage through a separate mechanism. Although we searched for pleiotropy using the MR‐PRESSO global test and MR‐Egger regression, we were unable to detect it. Finally, a larger study is required to identify and examine potential processes linking UC and ICH risk.

## 5 CONCLUSION

There is a causal relationship between UC and the risk of ICH even while IBD and UC did not. Future research needs to go into great detail on the mechanism through which UC influences ICH.

## AUTHOR CONTRIBUTIONS

Yanju Song and Xinfa Mao designed the research and decided on the manuscript's structure. Xuelun Zou and Yanju Song chose the references and participated in the writing. Yanju Song compiled the GWAS data. Yanju Song contributed to the analysis of these Mendelian randomization results. Xinfa Mao, Yi Zeng, and Le Zhang contributed to the manuscript's revision and completion. All authors participated in and approved the final draft of the manuscript.

## CONFLICT OF INTEREST STATEMENT

The authors declare no conflict of interest.

## Supporting information

Forest plot for this study in IIBDGC databases.

Supporting information.

## Data Availability

Requests for any data can be made to the authors.

## References

[1] D'Silva A , Fox DE , Nasser Y , et al. Prevalence and risk factors for fatigue in adults with inflammatory bowel disease: a systematic review with meta‐analysis. Clin Gastroenterol Hepatol. 2022;20(5):995‐1009. 10.1016/j.cgh.2021.06.034 34216824

[2] Nielsen OH , Gubatan JM , Juhl CB , Streett SE , Maxwell C . Biologics for inflammatory bowel disease and their safety in pregnancy: a systematic review and meta‐analysis. Clin Gastroenterol Hepatol. 2022;20(1):74‐87. 10.1016/j.cgh.2020.09.021 32931960

[3] Bigeh A , Sanchez A , Maestas C , Gulati M . Inflammatory bowel disease and the risk for cardiovascular disease: does all inflammation lead to heart disease? Trends Cardiovasc Med. 2020;30(8):463‐469. 10.1016/j.tcm.2019.10.001 31653485

[4] Xiong Z , Peng K , Song S , et al. Cerebral intraparenchymal hemorrhage changes patients' gut bacteria composition and function. Front Cell Infect Microbiol. 2022;12:829491. 10.3389/fcimb.2022.829491 35372117 PMC8966894

[5] Honarpisheh P , Bryan RM , McCullough LD . Aging microbiota‐gut‐brain axis in stroke risk and outcome. Circ Res. 2022;130(8):1112‐1144. 10.1161/CIRCRESAHA.122.319983 35420913 PMC9674376

[6] Chen Y , Wang X . Increased risk of stroke among patients with inflammatory bowel disease: a PRISMA‐compliant meta‐analysis. Brain Behav. 2021;11(6):e02159. 10.1002/brb3.2159 33960728 PMC8213927

[7] Alayo QA , Loftus Jr., EV , Yarur A , et al. Inflammatory bowel disease is associated with an increased risk of incident acute arterial events: analysis of the United Kingdom Biobank. Clin Gastroenterol Hepatol. 2023;21(3):761‐770. 10.1016/j.cgh.2022.08.035 36075499

[8] Huang WS , Tseng CH , Chen PC , et al. Inflammatory bowel diseases increase future ischemic stroke risk: a Taiwanese population‐based retrospective cohort study. Eur J Intern Med. 2014;25(6):561‐565. 10.1016/j.ejim.2014.05.009 24906568

[9] Wang S , Zou XL , Wu LX , et al. Epidemiology of intracerebral hemorrhage: a systematic review and meta‐analysis. Front Neurol. 2022;13:915813. 10.3389/fneur.2022.915813 36188383 PMC9523083

[10] GBD . 2019 Stroke Collaborators. Global, regional, and national burden of stroke and its risk factors, 1990‐2019: a systematic analysis for the Global Burden of Disease Study 2019. Lancet Neurol. 2021;20(10):795‐820. 10.1016/S1474-4422(21)00252-0 34487721 PMC8443449

[11] Krishnamurthi RV , Ikeda T , Feigin VL . Global, regional and country‐specific burden of ischaemic stroke, intracerebral haemorrhage and subarachnoid haemorrhage: a systematic analysis of the Global Burden of Disease Study 2017. Neuroepidemiology. 2020;54(2):171‐179. 10.1159/000506396 32079017

[12] Lamina C . Mendelian randomization: principles and its usage in Lp(a) research. Atherosclerosis. 2022;349:36‐41. 10.1016/j.atherosclerosis.2022.04.013 35606074

[13] Zou X , Wang L , Zeng Y , Zhang L . Illuminating the potential causality of serum level of matrix metalloproteinases and the occurrence of cardiovascular and cerebrovascular diseases: a Mendelian randomization study. J Hum Genet. 2023;68(9):615‐624. 10.1038/s10038-023-01154-0 37106065

[14] Liu JZ , van Sommeren S , Huang H , et al. Association analyses identify 38 susceptibility loci for inflammatory bowel disease and highlight shared genetic risk across populations. Nat Genet. 2015;47(9):979‐986. 10.1038/ng.3359 26192919 PMC4881818

[15] Woo D , Falcone GJ , Devan WJ , et al. Meta‐analysis of genome‐wide association studies identifies 1q22 as a susceptibility locus for intracerebral hemorrhage. Am J Hum Genet. 2014;94(4):511‐521. 10.1016/j.ajhg.2014.02.012 24656865 PMC3980413

[16] Zou X , Zhang L , Wang L , Wang S , Zeng Y . Exploring the causality of type 1 diabetes and stroke risk: a Mendelian randomization study and meta‐analysis. Mol Neurobiol. Published online July 26, 2023. 10.1007/s12035-023-03517-2 37493922

[17] Bowden J , Del Greco M F , Minelli C , Davey Smith G , Sheehan N , Thompson J . A framework for the investigation of pleiotropy in two‐sample summary data Mendelian randomization. Stat Med. 2017;36(11):1783‐1802. 10.1002/sim.7221 28114746 PMC5434863

[18] Bowden J , Davey Smith G , Haycock PC , Burgess S . Consistent estimation in Mendelian randomization with some invalid instruments using a weighted median estimator. Genet Epidemiol. 2016;40(4):304‐314. 10.1002/gepi.21965 27061298 PMC4849733

[19] Bowden J , Del Greco M F , Minelli C , Davey Smith G , Sheehan NA , Thompson JR . Assessing the suitability of summary data for two‐sample Mendelian randomization analyses using MR‐Egger regression: the role of the I2 statistic. Int J Epidemiol. 2016;45(6):1961‐1974. 10.1093/ije/dyw220 27616674 PMC5446088

[20] Zhao Q , Wang J , Hemani G , Bowden J , Small DS . Statistical inference in two‐sample summary‐data Mendelian randomization using robust adjusted profile score. Ann Statist. 2020;48(3):1742‐1769. 10.1214/19-aos1866

[21] Verbanck M , Chen CY , Neale B , Do R . Detection of widespread horizontal pleiotropy in causal relationships inferred from Mendelian randomization between complex traits and diseases. Nat Genet. 2018;50(5):693‐698. 10.1038/s41588-018-0099-7 29686387 PMC6083837

[22] Zou X , Wang L , Wang S , Zhang L . Mendelian randomization study and meta‐analysis exploring the causality of age at menarche and the risk of intracerebral hemorrhage and ischemic stroke. CNS Neurosci Ther. 2023;29(10):3043‐3052. 10.1111/cns.14245 37170723 PMC10493675

[23] Zöller B , Li X , Sundquist J , Sundquist K . Risk of subsequent ischemic and hemorrhagic stroke in patients hospitalized for immune‐mediated diseases: a nationwide follow‐up study from Sweden. BMC Neurol. 2012;12(12):41. 10.1186/1471-2377-12-41 22708578 PMC3430565

[24] Wu H , Hu T , Hao H , Hill MA , Xu C , Liu Z . Inflammatory bowel disease and cardiovascular diseases: a concise review. Eur Heart J Open. 2021;2(1):oeab029. 10.1093/ehjopen/oeab029 35919661 PMC9242064

[25] Mitsialis V , Wall S , Liu P , et al. Single‐cell analyses of colon and blood reveal distinct immune cell signatures of ulcerative colitis and Crohn's disease. Gastroenterology. 2020;159(2):591‐608. 10.1053/j.gastro.2020.04.074 32428507 PMC8166295

[26] Shen J , Ran ZH , Zhang Y , et al. Biomarkers of altered coagulation and fibrinolysis as measures of disease activity in active inflammatory bowel disease: a gender‐stratified, cohort analysis. Thromb Res. 2009;123(4):604‐611. 10.1016/j.thromres.2008.04.004 18499234

[27] Wang X , Chen S , Xiang H , et al. S1PR2/RhoA/ROCK1 pathway promotes inflammatory bowel disease by inducing intestinal vascular endothelial barrier damage and M1 macrophage polarization. Biochem Pharmacol. 2022;201:115077. 10.1016/j.bcp.2022.115077 35537530

[28] Zhang H , Wang X . Risk factors of venous thromboembolism in inflammatory bowel disease: a systematic review and meta‐analysis. Front Med. 2021;28(8):693927. 10.3389/fmed.2021.693927 PMC8273255 34262920

[29] Gu YH , Hawkins BT , Izawa Y , Yoshikawa Y , Koziol JA , Del Zoppo GJ . Intracerebral hemorrhage and thrombin‐induced alterations in cerebral microvessel matrix. J Cereb Blood Flow Metab. 2022;42(9):1732‐1747. 10.1177/0271678.221099092 35510668 PMC9441730

