# Supplementary figures and images for: Inflammatory bowel disease and the risk of intracerebral hemorrhage: A Mendelian randomization study and meta‐analysis
Source: Immun Inflamm Dis. 2023 Oct 17;11(10):e1048. doi: 10.1002/iid3.1048 (PMC10580698; doi:10.1002/iid3.1048)

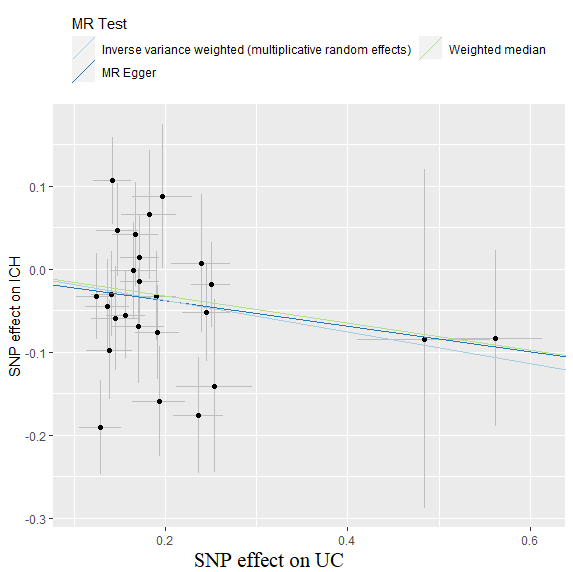

Supplement: Supplementary file 1 — Forest plot for this study in IIBDGC databases. [file IID3-11-e1048-s002.tiff]
